# Axonal Dynamics of Excitatory and Inhibitory Neurons in Somatosensory Cortex

**DOI:** 10.1371/journal.pbio.1000395

**Published:** 2010-06-15

**Authors:** Sally A. Marik, Homare Yamahachi, Justin N. J. McManus, Gabor Szabo, Charles D. Gilbert

**Affiliations:** 1 Laboratory of Neurobiology, The Rockefeller University, New York, New York, United States of America; 2 Institute of Experimental Medicine, Budapest, Hungary; University of California Berkeley, United States of America

## Abstract

Cortical topography can be remapped as a consequence of sensory deprivation, suggesting that cortical circuits are continually modified by experience. To see the effect of altered sensory experience on specific components of cortical circuits, we imaged neurons, labeled with a genetically modified adeno-associated virus, in the intact mouse somatosensory cortex before and after whisker plucking. Following whisker plucking we observed massive and rapid reorganization of the axons of both excitatory and inhibitory neurons, accompanied by a transient increase in bouton density. For horizontally projecting axons of excitatory neurons there was a net increase in axonal projections from the non-deprived whisker barrel columns into the deprived barrel columns. The axon collaterals of inhibitory neurons located in the deprived whisker barrel columns retracted in the vicinity of their somata and sprouted long-range projections beyond their normal reach towards the non-deprived whisker barrel columns. These results suggest that alterations in the balance of excitation and inhibition in deprived and non-deprived barrel columns underlie the topographic remapping associated with sensory deprivation.

## Abstract 2

Electrophysiology-delivery of fluorescent viral vectors-and two-photon microscopy were used to demonstrate the rapidity of axonal restructuring of both excitatory and inhibitory neurons in rodent cortical layer II/III following alterations in sensory experience.

## Author Summary

The adult brain is capable of learning new tasks and being shaped by new experiences. Evidence for experience-dependent plasticity of the adult cerebral cortex is seen in the functional rearrangement of cortical maps of sensory input and in the formation of new connections following alteration of sensory experience. The barrel cortex of the rodent receives sensory input from the whiskers and is an ideal model for examining the influence of experience on cortical function and circuitry. In the current study, we asked how experience alters cortical circuitry by examining excitatory and inhibitory axons within the adult whisker barrel cortex before and after plucking of a whisker and hence removal of its sensory input. By combining delivery of genes encoding fluorescent proteins, under the control of cell-type specific promoters, with two-photon imaging, we were able to directly examine subpopulations of axons and to determine when and to what extent experience altered specific connections in the adult living brain. Following whisker plucking we observed both the retraction of existing connections and an exuberant amount of growth of new axons. Axonal restructuring occurred rapidly and continued to undergo changes over the following weeks, with reciprocal sprouting of axons of excitatory neurons located in non-deprived cortex and of inhibitory neurons located in deprived cortex. The changes in the inhibitory circuits preceded those seen for excitatory connections.

## Introduction

The adult cortex adapts to alterations in sensory experience. This experience-dependent plasticity is evidenced by the functional reorganization of the primary sensory maps of the brain [1]–[12], synaptogenesis in the adult brain [13]–[16], and reorganization of dendrites [17],[18]. Detailed knowledge of the structural rewiring of cortical circuitry following sensory loss provides insight into the operation of cortical circuits: which circuits are involved in specific functions, how they can be altered by sensory deprivation and learning, and how they reorganize following nervous system damage (e.g., retinal lesions, stroke, neurodegenerative disease or amputation). With the availability of genetically engineered viruses and two-photon microscopy, we are able to examine, in the living animal, how cortical circuits are modified by sensory experience.

Cortical reorganization may be mediated through both excitatory and inhibitory connections. Pyramidal neurons in the superficial cortical layers form long-range horizontally projecting axons, which undergo a process of sprouting and synaptogenesis that parallels the functional reorganization of the cortex following altered sensory experience [6],[13],[14],[19],[20]. While the role of these excitatory cells in cortical reorganization has received the most attention, inhibition is also known to regulate plasticity during the critical period in early postnatal development [21]. Locally projecting inhibitory interneurons, also present in layer 2/3, comprise 20%–25% of all cortical neurons [22],[23]. The presence of inhibitory responses establishes the beginning of the critical period [24],[25], while the end of the critical period is dependent on inhibitory interneuron maturation [26]. Given its role in regulating plasticity during development, inhibition may also play a critical part in experience-dependent plasticity in the adult. A reduction in inhibition could unmask a network of normally subthreshold horizontal connections, driving their influence above threshold as a consequence of sensory deprivation.

The rodent whisker-barrel system is a classic model system to study the effects of experience and sensory loss on neural circuitry. During early development, whisker deprivation disrupts the barrel structure in primary somatosensory cortex (S1) [1]. Whisker ablation as early as P7 induces an expansion of the representation of non-deprived whiskers into the deprived barrel columns [27]. Although gross morphological changes in layer 4 barrel structure may have a critical period, the ability to induce changes in the cortical topography persists in the adult barrel cortex [3],[28],[29]. Notably, layers 2/3 and 5 retain the ability to undergo plasticity throughout life.

In the current study, we investigated the nature and time course of alteration of axonal arbors of different neuronal classes following whisker plucking in adult animals. We compared the patterns of remodeling of excitatory connections, formed by intrinsic long range horizontal projection of pyramidal neurons, with those of locally projecting inhibitory interneurons. We labeled subsets of neurons with one of two adeno-associated viruses (AAVs) carrying the enhanced green fluorescent protein (eGFP) or enhanced yellow fluorescent protein (eYFP) gene under the control of different promoters. We then imaged the labeled axons once before, and multiple times following, whisker plucking. By combining receptive field mapping, viral injections, and two-photon imaging, we were able to determine the location of the labeled somata and axons in reference to the somatosensory map. We could then directly observe the structural changes of the two main populations of layer 2/3 neurons within the deprived and non-deprived barrel columns over time and determine the extent of structural plasticity following sensory loss. Our studies show reciprocal changes in the long-range horizontal excitatory connections and inhibitory connections within layer 2/3 that parallel experience-dependent reorganization of the somatotopic map.

## Results

**Figure 1:**
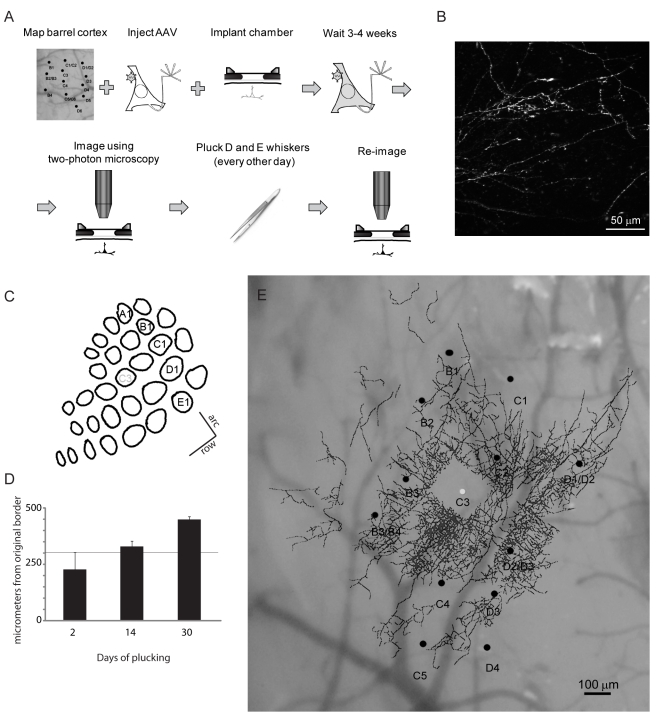
Methods for longitudinal imaging of cortical circuitry. (A) Timeline of experimental procedures. The barrel cortex is mapped electrophysiologically in order to guide placement of injections of genetically modified virus. After allowing several weeks for expression of genes carried by the virus, images of fluorescent label are taken to establish baseline connectivity. The cortex is reimaged at various time points following initiation of whisker plucking. (B) Surface view of horizontally projecting axons, imaged with 2-photon microscopy. (C) Cortical representation of mouse whiskers, with barrels arranged in rows (A to E) and arcs (1 to 6). (D) Whisker plucking induced remapping of barrel cortex topography in adult animals. The movement of the representation of C row whiskers into the deprived cortex following plucking of D and E rows is plotted as a function of the number of days of plucking. The original C/D row border corresponds to zero on the *y*-axis. The horizontal line at 300 micrometers indicates the original boundary between the D and E rows. (E) A reconstruction of the axonal plexus of excitatory neurons labeled by AAV-eYFP injection into C3 barrel column, shown in surface view. The axonal reconstruction is superimposed on the whisker map. The virus injection site was at the center of the C3 barrel column.

**Figure 2:**
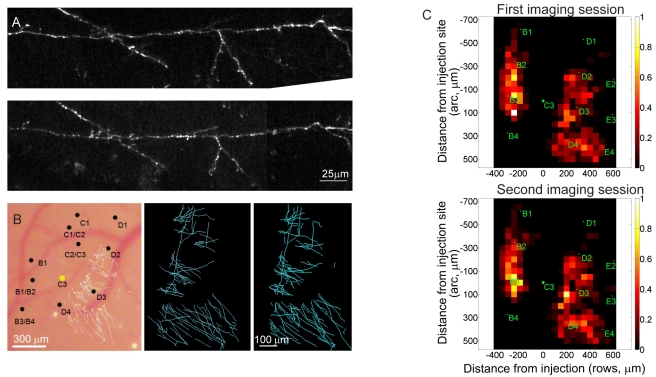
Axons and synaptic boutons during normal experience. (A) Axons at initial imaging session (top) and 1 wk later (bottom). (B) Axonal arbors imaged at two time points. Left, map of the whisker representation superimposed with the reconstruction of axons projecting toward rows D and E. Middle, reconstruction of the long-range horizontal axons imaged the first session. And right, 1 wk later. (C) Distribution of axonal density surrounding the injection site, averaged over all the control animals. Bins are spaced at 100 µm intervals with the injection site at (0,0,0). Axons immediately surrounding injection are not shown. Top, first imaging session. Bottom, second imaging session.

To label different components of the cortical circuit, we used a virus, AAV, that was genetically modified to deliver the gene encoding either eGFP or eYFP. For one strain of virus, eYFP was placed under the control of a promoter that induces expression in all neurons, the cytomegalovirus (CMV) promoter. Another strain was engineered to provide selective expression in inhibitory neurons, by putting the fluorescent gene under the control of the promoter for GAD65, the GABA synthetic enzyme. The time line of the experiment is shown in Figure 1A. The somatosensory cortex was mapped with a series of electrode penetrations in order to identify the barrel columns receiving input from specific whiskers. We then made a small (10 nl, CMV.eYFP.AAV; 20 nl for GAD65.eGFP.AAV) injection of a high titer (CMV.eYFP.AAV: 1×10^11^ particles per milliliter, GAD65.eGFP.AAV: 2×10^12^ particles per milliliter) virus into a selected barrel column. After making the injection, we allowed a period of time (3–4 wk) to elapse before the initial imaging session. This length of time is required to obtain full expression of the genes introduced with the viral vectors. We then acquired a series of about 30 slightly overlapping z-stacks, to cover a volume of cortex containing labeled neurons and their axons, approximately 1.1 mm×1.3 mm and 300 µm depth. All neurons and the full extent of their axons in the superficial layers of S1 were imaged. Each of the z-stacks consisted of ∼300 images, each separated by 1 µm in the z axis. After the initial imaging session, we plucked the D and E row whiskers, and continued to pluck them every other day, for periods of time ranging from 2 to 60 d (Figure 1A), after which we re-imaged the same cortical area. We also remapped the barrel cortex electrophysiologically to document any changes in the barrel map (Figure 1D). In a separate series of control animals, we reimaged the cortex over different periods in the absence of whisker plucking (Figure 2).

### Functional Modification in Barrel Cortex Following Whisker Plucking

First we confirmed that adult functional topography was stable over time in the absence of whisker plucking (unpublished data) and that our procedure for whisker plucking did induce remapping of cortical topography (Figure 1D). We mapped the cortex of 14 mice using the cortical vasculature as a fiducial reference for the recording sites. In animals with the whiskers left intact, we found no reorganization of the cortical topography 1 mo after the initial mapping (unpublished data). Following whisker plucking, however, and in agreement with previous studies, the cortical representation of non-deprived rows expanded into the deprived rows representing the plucked whiskers (Figure 1D). The cortical topography was remapped between 2 and 30 d of whisker plucking. The locations of cortical sites in the whisker barrel map were compared over the different time points. Consistently, the non-deprived C row expanded into the adjacent, deprived rows (Figure 1D).

### Pyramidal Neurons in Normal Experience

**Figure 3:**
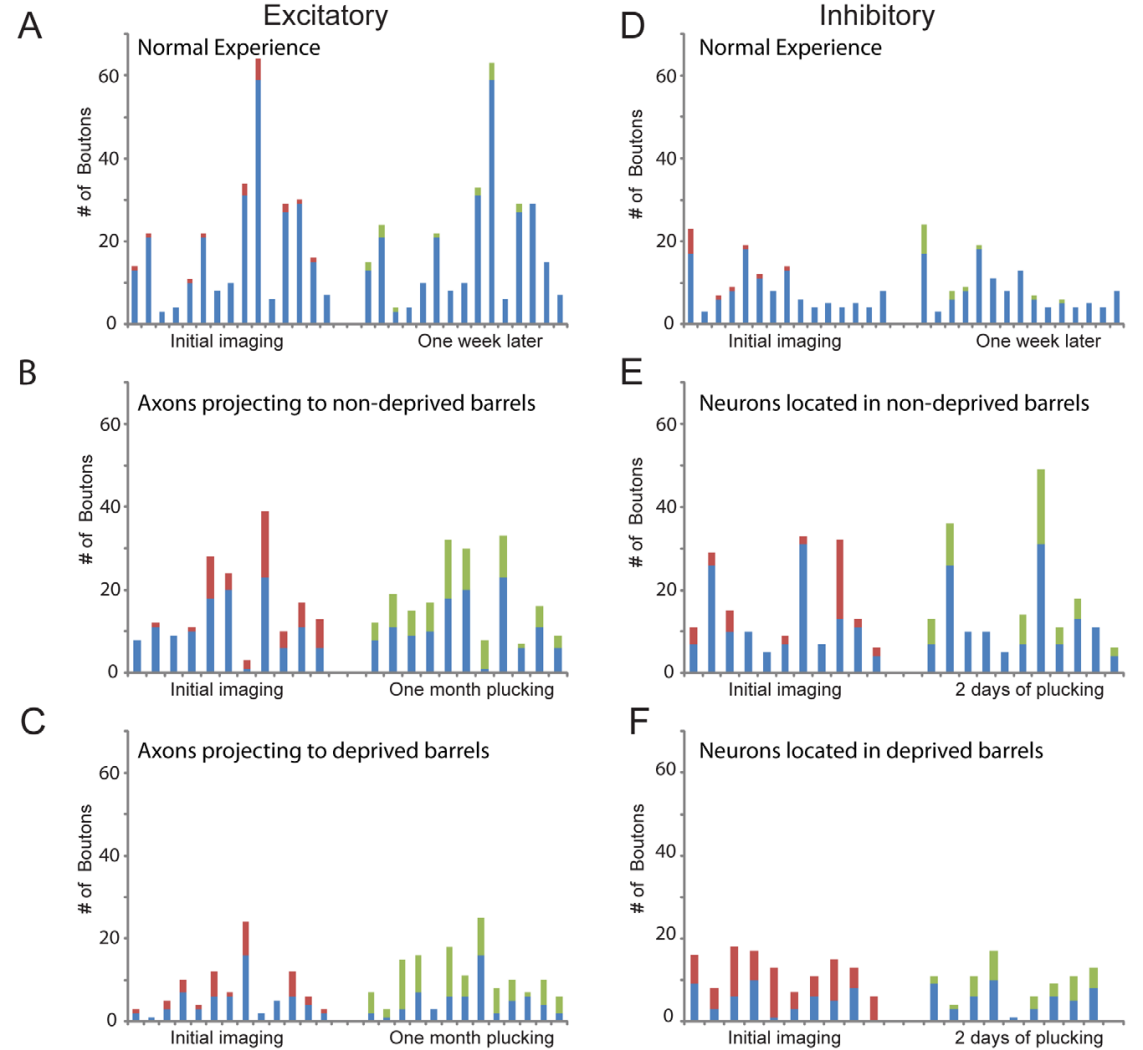
Bouton dynamics of selected axons in superficial cortical layers of whisker barrel columns of deprived and non-deprived animals. Graphs depict the number of boutons on axons before and after whisker plucking. Each bar represents a single identified axon, imaged before (left set) and the same axons imaged after whisker plucking (right set). Blue: stable boutons, red: eliminated boutons, green: added boutons. (A–C) Excitatory neurons. (A) Bouton turnover in whisker barrel columns in animals that underwent normal experience. (B) Bouton turnover during whisker plucking for axons projecting into non-deprived barrel columns and (C) projecting into deprived barrel columns (D–F) Inhibitory interneurons. (D) Bouton turnover in whisker barrel columns of animals that underwent normal experience. (E) Axonal boutons of neurons located in non-deprived barrel columns before and after whisker plucking. (F) Boutons of neurons located in deprived barrel columns.

To study the anatomical correlates of this functional reorganization, we first examined the dynamics of pyramidal cell axons during normal experience. The baseline reconstruction of the axonal plexus was done by imaging in and around the injection site (C3 barrel column). The same area, encompassing the extent of the axonal arbors of labeled neurons, was imaged at subsequent time points. Horizontal axons of layer 2/3 pyramidal neurons were identified by their characteristically long range axons, which run parallel to the cortical surface. We imaged mice under normal sensory experience over time periods extending for up to 1 mo. The plexus of long-range horizontal connections was reconstructed both for axons innervating rows A and B as well as rows D and E. There was no net sprouting or retraction of axon collaterals (Figure 2). The axonal boutons, on the other hand, showed significant change (Figure 3A), having rates of addition and disappearance of, respectively, 6±2.4% and 6±1.1% per week (mean ± S.E.M.), which closely correspond to rates observed across species and sensory systems [30],[31].

### Pyramidal Axons Projecting from Non-Deprived to Deprived Barrel Columns

**Figure 4:**
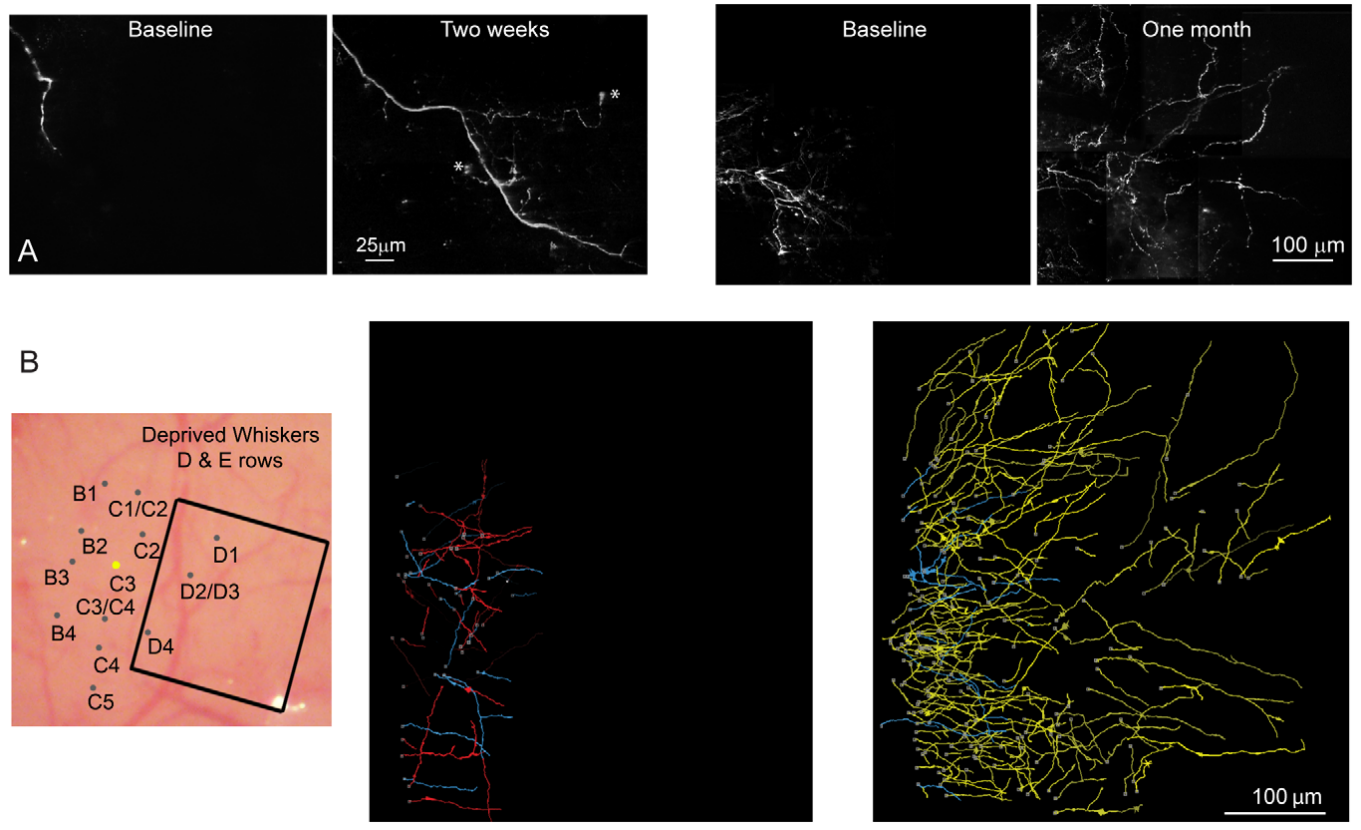
Axons of layer 2/3 excitatory neurons originating from C3 barrel column projecting to deprived barrel columns following whisker plucking. (A) Left: Montage of z-projections located within a deprived barrel column before (far left) and after (second from left) 14 d of whisker plucking. Right, Montage of z-projections of a cluster of axons located within deprived cortex both before (3^rd^ from left) and after (far right) 1 mo of whisker plucking. * - denotes putative growth cone. (B) Axons originating from C3 injection site projecting to rows D and E following whisker plucking. Left, the map of the barrel column field. The square box depicts the region shown in the two reconstructions to the right. Middle, axons located within D and E rows before plucking. Right, same area after 1 mo of plucking. Axons that were stable over the two imaging sessions are shown in blue; axons that retracted from the first to second imaging session are in red; new axons are plotted in yellow.

**Figure 5:**
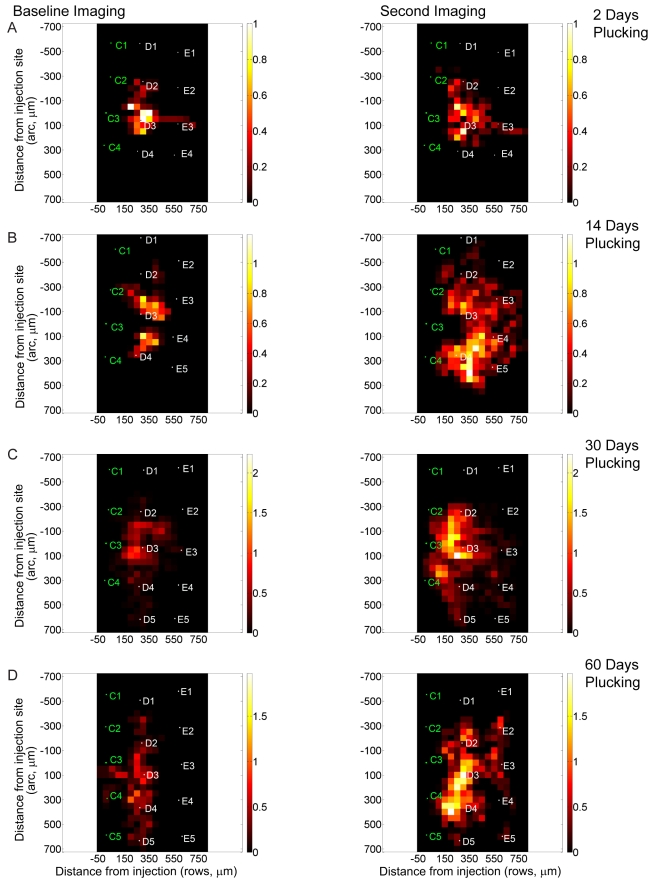
Changes in axonal density following varying durations of whisker plucking. (A–D) For 2 (A), 14 (B), 30 (C), and 60 (D) d. Left column, first imaging session. Right column, second imaging session. The axonal length in each bin was averaged over multiple animals and then normalized. The normalization was performed by dividing all the bins in each baseline condition by the value of the highest density bin maximum in that condition. The bins in the corresponding post-plucking time point were divided by the same number. The maximum mean axonal length for any bin in each baseline graph is therefore 1.0 (expressed in arbitrary units, A.U.), and the length in the corresponding post-plucking graph is expressed relative to that standard. The bins in the histogram are 50 µm×50 µm; the injection site is located at coordinate (0,0,0). Average location of barrel column for animals in each condition is indicated on each graph. White text depicts deprived barrel columns. Green text depicts non-deprived barrel columns.

We imaged the pattern of arborization of axons extending from normal to deprived cortex after variable periods of whisker plucking. Long-range axons underwent a massive reorganization, which included strong sprouting (yellow collaterals, Figure 4B) and weaker retraction (red collaterals, Figure 4B), resulting in a large net increase in density (Figures 4 and 5). The change in axonal density after 2 d of plucking was small (Figure 5A), but after 14 d, the axonal growth was exuberant, extending deeply into the deprived barrel columns (Figures 4A and 5B). At this time, putative growth cones were observed (Figure 4A, asterisk). Some of the added axonal arbor increased the range of the horizontal projections beyond their normal extent. After 14 d of plucking, the axons originating from non-deprived cortex had increased their density in the deprived barrel columns by a factor of 3.59 (Figures 4A and 5B). At every time point the sprouting of axons was accompanied by pruning, to a smaller degree, of a portion of the preexisting collaterals (30 d: 3.21±0.65 and 60 d: 3.0±0.37; mean ± S.E.M.; Figure 5C and 5D). The net effect of plucking, by the end of the imaging period, was a >3-fold net increase of density of horizontal axon collaterals in the deprived rows.

In order to examine bouton dynamics, we divided the boutons into two main groups based on whether they were located on axons that were stable over consecutive imaging sessions or if they were on dynamic axonal branches (e.g., axons that were added or lost following whisker plucking). During a period of 30 d of whisker plucking, boutons were dynamically recycled even on axons that were stable during both imaging sessions. After 30 d of plucking, 33±5% of the original boutons on stable axons were retracted, while 82±2.4% of the boutons were newly formed (Figure 3C). For the stable axons, therefore, bouton density increased and their rate of turnover also increased relative to that observed during normal experience.

### Pyramidal Axons Projecting between Non-Deprived Barrel Columns

**Figure 6:**
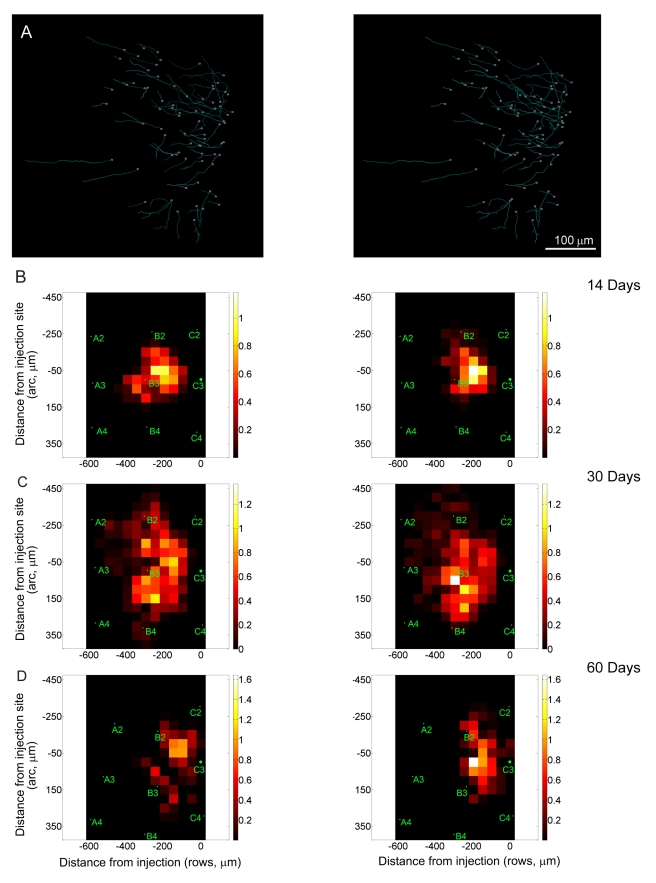
Excitatory axons originating from neurons within non-deprived barrel columns. (A) Excitatory axons located within non-deprived barrel columns. Left, axons located in rows A and B before whisker plucking. Right, same area following 1 mo of whisker plucking. (B–D) Axonal density in non-deprived cortex after 14 (B), 30 (C), and 60 (D) d of whisker plucking. Left column, first imaging session. Right column, second imaging session after the indicated period of plucking. As in Figure 5, the axonal density at each pair of time points was averaged over a set of mice and normalized by dividing each bin by the maximum region of local density in the baseline condition. Normalized mean axonal density is plotted (in arbitrary units) in 50 µm×50 µm bins with the respect to the injection site (asterisk) at coordinate (0,0,0). Distances are expressed in µm from injection site. Average location of barrel column for animals in each condition is indicated on each graph. Green labels refer to non-deprived barrel columns.

Axons that projected into non-deprived barrel columns, on the side of the injected barrel columns opposite to that of the deprived barrel columns, were also examined. There was no change in the topography of unplucked barrel column rows A and B, even though they, like the deprived rows D and E, receive inputs from row C. We investigated the axonal dynamics of inputs to non-deprived barrel columns and compared them to animals that underwent normal experience. There was no net change in the density of axons projecting towards non-deprived barrel columns (rows A and B) (Figure 6). They did, however, undergo minor additions to and pruning of their axonal arbors (Figure 6B–6D). Additionally, bouton turnover rate was higher than observed for control non-deprived animals. Boutons were added and eliminated at rates of 43±18.2% and 29±6.7% per month, respectively (Figure 3B). The number of boutons per micrometer increased from 0.09 (baseline) to 0.14 (following deprivation of D and E rows).

### Inhibitory Interneurons

The horizontal axons of excitatory neurons projecting into the deprived cortical region, which underwent massive restructuring and synaptogenesis following whisker deprivation, originate from pyramidal cells. We next turn to the role of inhibitory neurons in the reorganization. We imaged the axons of inhibitory interneurons before, and 2 to 30 d after whisker deprivation in either deprived or non-deprived barrel columns. In one animal, we imaged axons on an hourly basis following whisker plucking.

### Viral Vector to Label Inhibitory Interneurons

**Figure 7:**
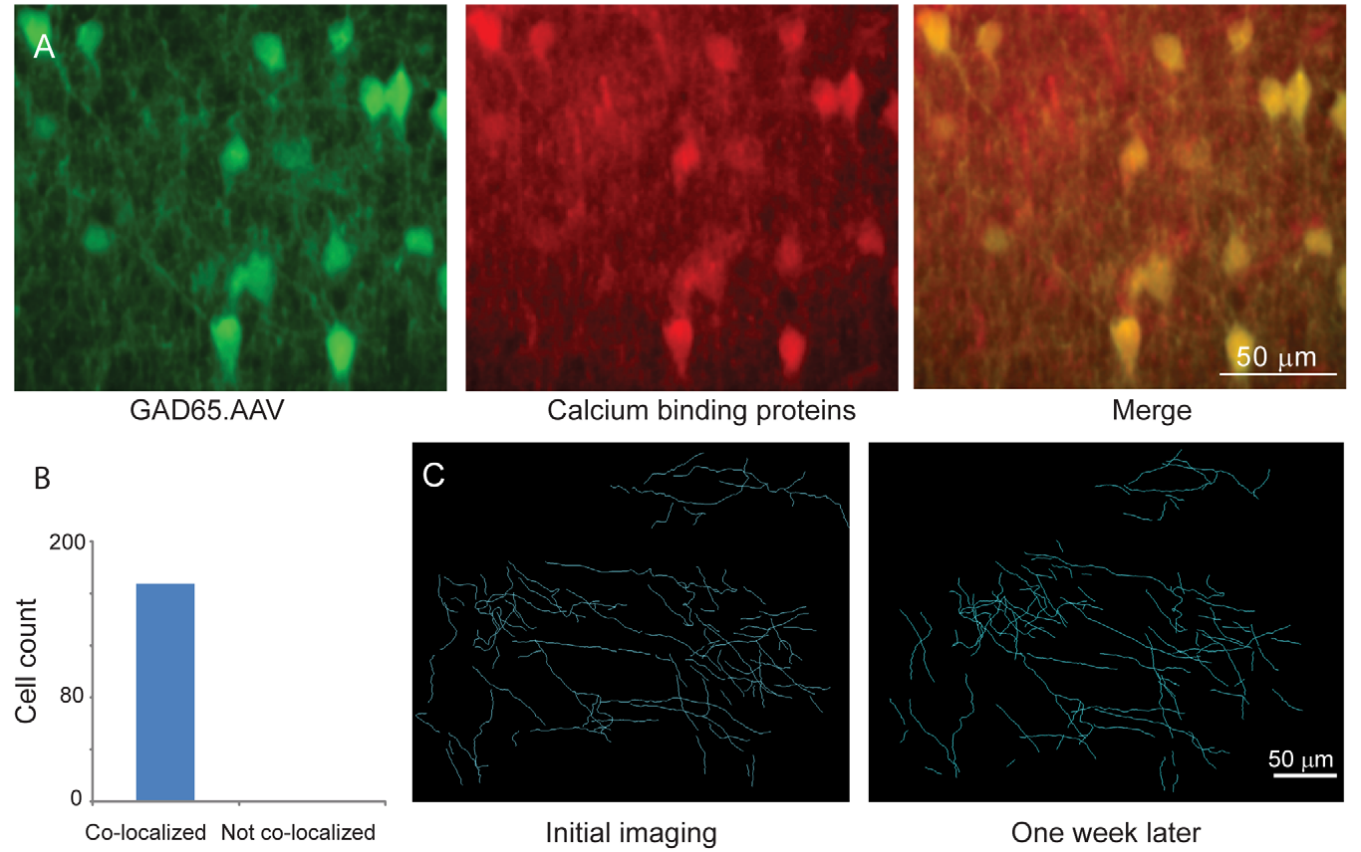
Cell type specificity of expression in GAD65.eGFP.AAV infected neurons. (A) Combined eGFP expression and labeling with antibodies against calcium-binding proteins. Left, neurons labeled with GAD65.eGFP.AAV. Middle, neurons labeled with a cocktail of antibodies against the calcium-binding proteins calbindin, calretinin, and parvalbumin. Right, merged images, double-labeled neurons are yellow. (B) Number of neurons labeled by GAD65.eGFP.AAV that were co-localized with calcium binding proteins. (C) Axons of inhibitory neurons in normal cortex. Left, initial imaging session. Right, same region imaged 5 d later.

In order to observe the structural dynamics of inhibitory interneurons, we genetically modified an AAV vector to label inhibitory interneurons exclusively by driving eGFP expression under the GAD65 promoter. We confirmed the selectivity of GAD65.eGFP.AAV by using antibodies against a combination of three calcium-binding proteins (calbindin, parvalbumin, and calretinin) that are expressed in 90% of almost completely non-overlapping groups of inhibitory interneurons [32]–[34]. All GFP expressing neurons were labeled with the antibody cocktail (Figure 7A–7B).

The GAD65.eGFP.AAV therefore allows us to observe the dynamics specifically for inhibitory cells. While the CMV promoter that was used to label long range excitatory connections labeled both interneurons and excitatory neurons, the long range connections that were the focus of the study were likely to originate primarily from excitatory neurons, since the axons of excitatory neurons are known to project much farther than the axons of inhibitory neurons. Since the longest range axons originate from excitatory cells, and since most axons imaged in the deprived barrel columns were far from the injection site, the experiments examining axonal dynamics with cells labeled with the CMV promoter were predominately excitatory axon collaterals. Additionally, as will be shown below, the inhibitory interneurons located in non-deprived cortex did not stretch their collaterals beyond their normal reach following deprivation, minimizing the potential amount of contamination of deprived barrel columns by inhibitory axons in the CMV.GFP injections. The longest range projections into the deprived barrel columns would therefore originate from excitatory neurons.

We injected the GAD65.eGFP.AAV virus in either rows B and C (non-deprived) or rows D and E (deprived) after the S1 barrel field was mapped electrophysiologically. We were able to directly observe the structural dynamics of inhibitory interneurons in both deprived and non-deprived cortex. As with the previous set of experiments, we plucked rows D and E completely at the end of the initial imaging session.

### Inhibitory Interneurons under Normal Experience

To establish the axonal dynamics of inhibitory interneurons in mice under normal experience, we imaged mice several times without whisker plucking. These animals did not exhibit significant changes in axonal density (Figure 7C). Boutons were added at 10±2.8% and retracted and 8±2% per week (Figure 3D). These rates were slightly higher than those observed for excitatory neurons.

### Inhibitory Interneurons in Non-Deprived Barrel Columns

**Figure 8:**
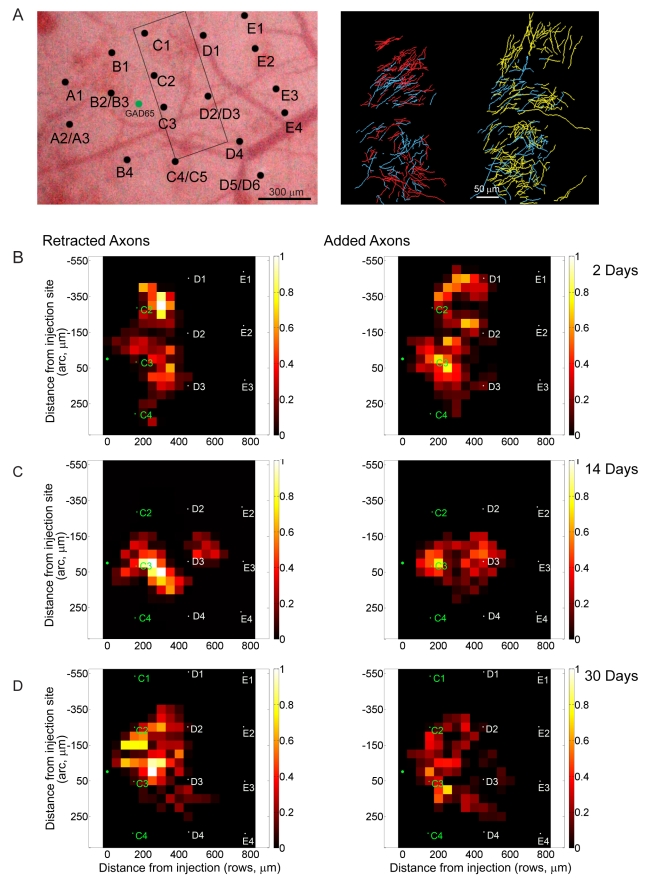
Axons of inhibitory interneurons located in the non-deprived rows following whisker plucking. (A) Top, the whisker barrel map. Rectangular box depicts the region of axonal reconstructions shown on the right. Middle, axons located within non-deprived rows before whisker plucking. Right, same area after 2 d of plucking. In the reconstruction, the axons that persisted over both sessions are shown in blue, axons retracted from the first to second imaging session in red, and new axons in yellow. Scale bar  = 50 µm. (B–D) Changes in axonal length for 2 (B), 14 (C), and 30 (D) d of whisker plucking. Left, the distribution of axonal length that was lost between each baseline and post-plucking time point. Right, the distribution of axonal length that was gained between the baseline and post-plucking time points. The data for each pair of time points were obtained by averaging over several mice. Here, the magnitude of the axonal changes for each data pair is normalized with respect to the maximum length of axon that was retracted within any bin. The maximum length of retracted axon in each data pair is therefore 1.0 (in arbitrary units of length), and the length of added axon is measured with respect to that value. The dimensions of the bins are 50 µm×50 µm. The average locations of barrel columns for animals in each condition are marked on each map, with deprived barrel columns indicated in white and non-deprived barrel columns indicated in green.

The inhibitory interneurons located within the non-deprived barrel columns underwent reorganization in the first days following whisker plucking (Figure 8). The process of structural reorganization was dynamic, with both axonal retraction and growth occurring throughout the axonal plexus, including the portions located within the deprived and non-deprived barrel columns. Unlike the excitatory axons originating from the non-deprived barrel columns, the overall envelope of the axonal field from non-deprived inhibitory neurons did not change significantly over a period of 30 d of plucking. We also examined the boutons of these neurons. Boutons appeared and disappeared at rates of 31±8.5% and 23±5.7% over a period of 2 d, respectively (Figure 3E).

### Inhibitory Interneurons in Deprived Barrel Columns

**Figure 9:**
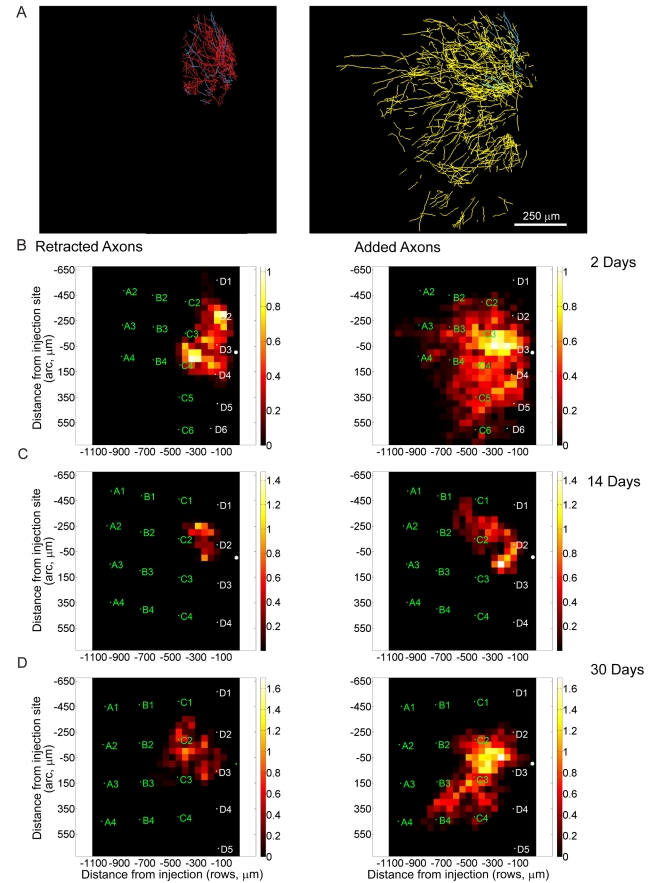
Axons of inhibitory interneurons located within deprived rows following 2 d of whisker plucking. (A) Left, axons located within deprived rows before plucking. Right, same area after 2 d of plucking. Axons that are stable over the two imaging sessions are color coded blue; those pruned after the first session colored red; and newly sprouted axons shown in yellow. (B–D) Quantification of changes in axonal length for 2 (B), 14 (C), and 30 (D) d of whisker plucking. As in Figure 8, the data are normalized relative to the highest density of retracted axons for each pair of time points. Retracted (left) and added (right) axons are plotted in 50 micron bins, with the injection site, shown by the asterisk, at coordinate (0,0,0). Distances are expressed in µm from injection site. The average locations of barrel columns for animals in each condition are marked on each graph. Deprived barrel column labels are in white, non-deprived barrel labels are in green.

**Figure 10:**
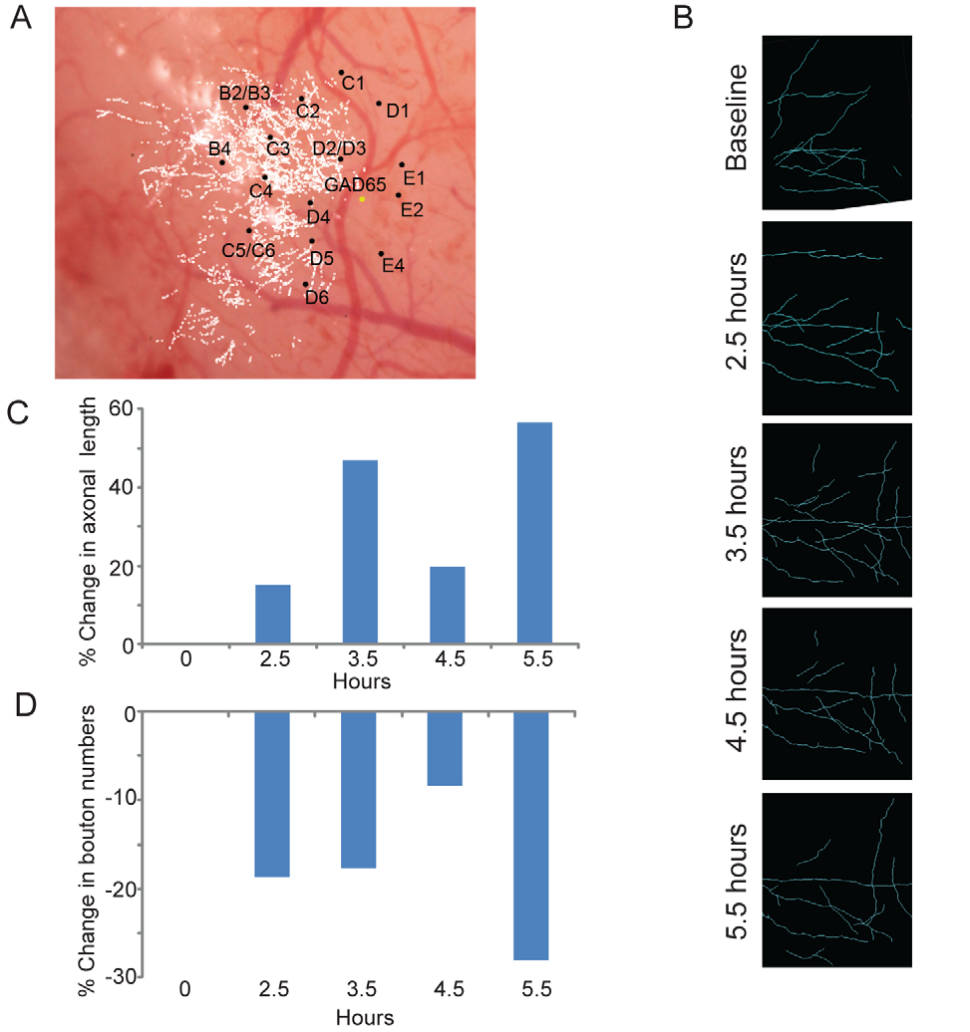
Short term changes in axonal length and bouton number. Over hours following the onset of plucking for inhibitory interneurons whose soma are located in deprived rows. (A) Location of boutons with respect to cortical topography. (B) Reconstruction of axons following whisker plucking. (C) Time course of change in axonal length (%). (D) Time course of change in bouton number (%).

The axonal arbors of inhibitory interneurons residing within the deprived barrel columns underwent massive reorganization (Figure 9). A large number of axons close to the cell somata retracted following 2 d of plucking. However, the most marked change was the increase in the lengths of axons extending into the cortical area surrounding the deprived barrel column after 2 d of plucking (Figure 9A–9B). At this time point total axonal length increased by a factor of 2.5. These axons, which typically sent axonal arbors approximately 450 µm from the injection site in normal cortex, now reached as far as 1,100 µm. Over the subsequent weeks there was a slight retraction in the overall extent of the inhibitory axon collaterals, but the new axons still extended well beyond their original reach. At 14 d and 30 d these axons reached all rows of whisker barrel columns and were stable (Figure 9C and 9D, right column). Interestingly, the new axons formed boutons almost exclusively around the outer perimeter of the barrel columns (Figure 10A). This distribution suggests that the newly formed inhibitory axons target either neurons located within the septal region of the barrel columns or the apical dendrites of layer V pyramids. These dendrites cluster along the barrel column walls as they reach towards the cortical surface [35].

In order to examine inhibitory interneuron bouton dynamics, we divided the axons into three main groups based on whether they were stable, added, or lost following whisker plucking. Turnover rates were determined for all of these categories. For the stable axons, bouton turnover was elevated following 2 d of whisker plucking, with bouton disappearance dominating over appearance (appearance: 26±5.3% and disappearance: 59±6.7% over the 2 d period; Figure 3F). This led to a net decrease in the density of boutons on stable axons (from 0.09 boutons/µm to 0.07 boutons/µm), which was the opposite of what we found for excitatory neurons. Inhibitory interneuron bouton density was 0.06 boutons/µm for axons destined for retraction and 0.05 boutons/µm for axons added following whisker plucking.

We also examined the axonal dynamics over shorter periods of whisker plucking by repeatedly imaging a short stack of axons located within the non-deprived cortex and originating from the deprived D and E rows. We imaged both before plucking and every hour following whisker plucking from 2.5 to 5.5 h (Figure 10B). At the earliest time point (2.5 h) axonal length increased (14.9%; Figure 10C) and bouton density decreased (−18.75%; Figure 10D). The axonal length and bouton numbers continued to change over the next few hours, involving both addition and retraction. At 5.5 h, we observed a net gain in axonal length of 56.5% while boutons were reduced by 28.1%.

**Figure 11:**
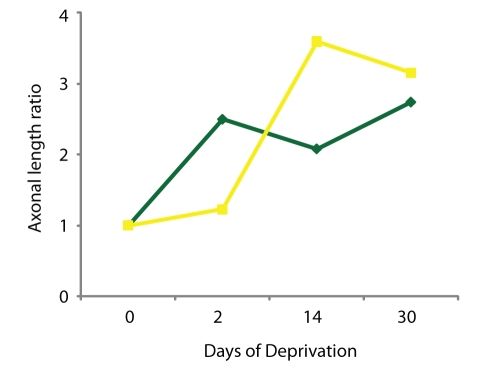
Change in axonal density plotted as a function of days of deprivation. Expressed as a ratio of axonal length at the indicated duration of deprivation to that measured before deprivation. The green line depicts axons of inhibitory interneurons whose somata are located in deprived barrel columns and are projecting into the non-deprived barrel columns. The yellow line depicts axons of excitatory neurons in the non-deprived barrel columns that are projecting into the deprived barrel columns.

We plotted the ratio of axonal length, for different durations of deprivation, over baseline before whisker plucking for axonal arbors of inhibitory interneurons residing within the deprived barrel columns and for axonal arbors of excitatory neurons residing in non-deprived cortex (Figure 11). Interestingly, the growth of inhibitory interneurons axons projecting to the non-deprived cortex preceded and increased more quickly than those of the excitatory neurons projecting into the deprived cortex. By 14 d of whisker plucking, however, excitatory axons surpassed the amount of growth seen for the inhibitory interneurons.

## Discussion

The combined techniques of labeling neurons with genes encoding fluorescent proteins, delivered with viral vectors, and longitudinal in vivo two-photon imaging enabled us to determine the dynamics of cortical circuitry following alterations in sensory experience. Moreover, the use of cell specific promoters allowed us to dissect out the contributions of different components of cortical circuits to the reorganization. Several novel features of the reorganization were revealed in these experiments. The axonal remodeling consisted of a parallel process of sprouting and pruning. While there was steady state turnover of both excitatory and inhibitory synapses in the absence of plucking, this turnover was dramatically elevated following sensory deprivation. Previous studies have focused primarily on the contribution of excitatory connections in the remodeling [16]–[18]. Here we observed striking changes in the axonal arbors of inhibitory neurons. The changes consisted not only of alterations in the density of axon collaterals in deprived and non-deprived barrel columns, but an extension of the envelope of axonal arborization far beyond its normal extent, both for excitatory neurons in non-deprived barrel columns and inhibitory neurons in deprived barrel columns. This contrasts with earlier findings that the axonal changes principally consisted of changes in the density of existing clusters of axon collaterals [13],[14]. Moreover, the changes observed in inhibitory interneurons located in the deprived barrel columns were extremely rapid and preceded the axonal growth of excitatory neurons from non-deprived cortex. Finally, while postmortem studies examined late time points after the onset of sensory deprivation, here we observed changes occurring very rapidly, in some instances within hours after the initial whisker plucking for inhibitory interneurons.

The reorganization of adult cortical topography has been detailed in many systems. Remapping occurs almost immediately and continues to progress in the weeks and months following peripheral lesions [36],[37]. Almost immediately following sensory loss, large regions within the deprived area become unresponsive [7],[9]. This is accompanied by an immediate expansion of the cortical representation of non-deprived sensory input into the deprived regions. The newly remapped sites have neurons with receptive fields that are larger than normal and that respond more sluggishly. Over time, remapping progresses, with the region of unresponsive cortex getting smaller and the new receptive fields shrinking back to their normal size [9]. In the model system we have used here, cortical reorganization following whisker plucking occurred across barrel columns. Previous studies have demonstrated topographic changes in the whisker barrel system within three and a half days of whisker trimming [28]. We observed the process of axonal remodeling of inhibitory interneurons to begin even earlier, within hours of whisker plucking. By carrying information across adjacent whisker barrel columns, the horizontally projecting axons are likely to represent the circuit mechanism of topographic remapping. Although one may have expected that short term functional changes would be attributed to alteration in the strength of existing synapses rather than axonal sprouting and synaptogenesis, the rapidity and massive onset of the morphological changes we observed opens the possibility that axonal remodeling may underlie even the earliest phases of cortical remapping.

Following whisker plucking, inhibitory interneurons from within the deprived cortex extended their axons into the non-deprived rows with extreme rapidity. Axons from excitatory neurons located in the non-deprived barrel columns invaded the deprived row on a slightly slower time course (Figure 11). Previous studies have demonstrated that cortical areas undergoing sensory deprivation show a transient decrease in GAD expression [38]–[40], while over-stimulated cortical areas show a very early yet transient upregulation of GAD, GABA, and/or GABA_A_R in the hours and days following the manipulation [41]–[43]. The retraction of inhibitory axons within the deprived barrel columns parallels the decrease in GAD expression. The observed decrease in bouton numbers following plucking may account for the decreased levels of GAD expression.

In the current study, the axonal arbors from inhibitory interneurons were as dynamic as excitatory axons following sensory deprivation. The extension of inhibitory axons from deprived to non-deprived cortex may represent a compensatory mechanism required to maintain the balance between excitation and inhibition that exists in normal cortex. We hypothesize that this regulation may require contacting the somata of the excitatory neurons that sprout into the deprived barrel columns, which would explain the extension of inhibitory axon collaterals into the non-deprived barrel columns. The functional match between inhibitory and excitatory tuning has been seen in orientation tuning in visual cortex [44],[45] and in frequency tuning in auditory cortex [46]–[48]. In order to maintain this balance when excitatory neurons extend their arbors into new territory, there appears to be a compensatory attraction of inhibitory axons towards these excitatory neurons.

Increasing evidence points towards an ongoing process of synaptogenesis and synapse elimination in the adult brain, even in the absence of deprivation [16],[30],[31],[49]. Our current results support these findings, with excitatory axons showing a constant rate of bouton turnover of 6% per week. Here we showed that inhibitory interneurons have a similarly high rate of turnover (8% to 10% per week). With altered sensory experience, the rate of bouton turnover accelerated several-fold. The largest rates of turnover were seen for the excitatory axons projecting into deprived cortex and for inhibitory axons projecting from deprived to non-deprived cortex.

Our results demonstrate that rapid alteration of cortical circuits accompanies the functional changes associated with sensory deprivation in the adult. The changes of inhibitory connections were as substantial as those seen for excitatory connections, and these results show that inhibition plays a key role in adult cortical plasticity, mirroring its role in early postnatal development. Although the changes we observed in this study are associated with sensory deprivation, similar mechanisms may apply to normal processes of experience-dependent change, such as those associated with perceptual learning.

## Methods

### Preparation of the Virus

We genetically engineered two AAVs to label subsets of neurons. One AAV contained the CMV promoter and the eYFP gene. The eYFP gene (derived from pEYFP-N1, Clontech) was PCR-amplified and then cloned into the pCMV-MCS Vector (Stratagene). The transgene was flanked by the two inverted terminal repeats. The vector was verified by sequencing. The AAV was prepared by packaging the vector plasmid with the AAV serotype 2/1 using a calcium phosphate transfection. The virus was purified using heparin affinity chromatography [50] and concentrated (Millipore Biomax 100K filter). Titer was determined by quantitative PCR using CMV-specific primers. The titer was determined to be 10^11^ viral particles per milliliter. The other AAV labeled inhibitory interneurons (GAD65.eGFP.AAV). The GAD65 promoter [51] was isolated from a plasmid that included 5.5 kilobases upstream from the start codon of GAD65 (provided by G. Szabo, Institute of Experimental Medicine of the Hungarian Academy of Sciences). From that 5.5 kb section, a 2.7 kb fragment directly upstream from the start codon of the GAD65 gene was amplified out via PCR; KpnI and AgeI restriction sites were added to the 5′ and 3′ sites, respectively. The KpnI primer sequence was 5′ CGAGGTACCAAGTAAGCAGAGGGGCAGTG; the AgeI sequence was 5′ CGAACCGGTGCAGAGCCATCTTCAGATCC. The GAD65 promoter sequence was inserted into a custom AAV2 plasmid (provided by J. Pena, The Rockefeller University). The AAV2 vector and the GAD65 promoter sequence were digested with Kpn I and Age I restriction enzymes. The GAD65 promoter was then cloned into the AAV2 vector, which contains the eGFP sequence and woodchuck hepatitis post-translational regulatory element (WPRE). The total resulting size of the AAV genome in the plasmid, including the region flanked by the ITR, is 4.7 kb. The titer was determined to be 2×10^13^ particles per millimeter by quantitative PCR using GFP-specific primers.

### Checking Specificity of GAD65.eGFP.AAV

Calbindin, calretenin, and parvalbumin are calcium-binding proteins that are expressed in almost non-overlapping populations of inhibitory interneurons. 90% of all inhibitory interneurons express one or another of these three proteins [32]–[34]. Antibodies for all three of these proteins were used to confirm the specificity of the virus. Animals were perfused with 2% paraformaldehyde in PBS and cryoprotected in 30% sucrose. The region of cortex including the injection site was sectioned at 30 µm. Sections were rinsed three times in PBS and incubated in a blocking media that contained 10% normal goat serum in Tris buffer solution (pH 7.4), 0.2% Triton X-100, for 1 h at room temperature. Primary antibodies were incubated for 48 h at 4°C. Primary antibodies: rabbit anti-calbindin (1∶5000, Swant), rabbit anti-calretinin (1∶2000, Swant), and rabbit anti-parvalbumin (1∶5000, Swant) were used. Sections were rinsed three times in tris buffer solution. Sections were then incubated with a secondary antibody: Cy3 Goat Anti-Rabbit (1∶500, Jackson ImmunoResearch laboratories) at room temperature for 2 h. Sections were rinsed three times before being mounted and coverslipped with 4% n-propyl gallate (Sigma-Aldrich) in 90% glycerol to prevent photo-bleaching.

### Surgery

All procedures were done according to institutional and federal guidelines. All mice (*N* = 42) used for these experiments were adult, 2 mo or older, at the time of the virus injection. Imaging began 3 to 4 wk later. They were anesthetized with ketamine (80 mg/kg) and xylazine (6 mg/ml). Dexamethasone (0.2 mg/Kg) was administered. A craniotomy was performed over the barrel cortex, but the dura was left intact. The receptive fields of the whiskers were mapped electrophysiologically. Recordings were taken between 300 and 500 µm below the cortical surface, in response to stimulation by whisker reflection with a rod. Receptive fields were mapped by using insulated tungsten microelectrodes (impedance 1–2 MΩ, Alpha Omega, Israel). Cortical spikes were acquired with an acquisition program (Plexon Inc, Dallas, TX), amplified 10,000 times, and fed into an audio monitor (Grass Medical Instruments, West Warwick, RI). An image of the exposed cortex was taken. Blood vessels were used to determine the location of the electrode in the cortex. Responses to electrode penetrations were recorded and labeled on the image using Photoshop CS (Adobe Systems Inc, San Jose, CA). The cortical topography was determined for the entire whisker barrel cortex. The functional and anatomical maps were brought into register by aligning the blood vessels in the cortical topography photomicrograph and the two photon images. Recording positions were established relative to the cortical surface vasculature, and the resulting map was used as the basis for the viral injections. The injections consisted of 10 nl of CMV.eYFP.AAV (1×10^11^ particles/ml) placed in the C3 barrel column, or 20 nl of GAD65.eGFP.AAV (2×10^13^ particles/ml), placed into either the deprived columns or non-deprived barrel columns. One of the two high-titer preparations of AAV was pressure injected into the cortex using borosilicate glass micropipettes (World Precision Instruments, Inc, Sarasota, FL) and Picospritzer III (Parker Hannifin Corp, Cleveland, OH). Several pulses at 0.1 bar were given at a depth of 350 micrometers over 1 min, with a 5 min resting period afterwards. Dura was left intact throughout the procedure. Agar and a 5 mm circular glass coverslip were placed over the craniotomy and sealed with dental acrylic (Lang Dental Manufacturing Co Inc, Wheeling, IL). Imaging began at least 3 wk following the viral injection to ensure full expression of the virus.

### Imaging

Animals were anesthetized with isoflurane (3% induction; 1.5%–2% maintenance). The cranial window was cleaned but the dura was left intact and the area was imaged with the 2-photon microscope. The labeled neurons and their axons were first imaged 3 to 4 wk following the viral injection to ensure full expression of the fluorescent protein. Following the baseline imaging session(s), whiskers from rows D and E were plucked every other day to prevent new whisker growth and thereby to maintain deprivation of the D and E whisker barrel columns. Imaging was done for variable intervals following the onset of the deprivation period.

Images were collected on a custom built 2-photon microscope that was modified from a Leica TCS Sp2 confocal microscope (Mannheim, Germany) with a custom moveable scanning head, which can be moved in three dimensions using a Sutter (Novato, CA) MP-285-3Z micromanipulator. The laser source was provided by a Ti-sapphire laser (Tsunami/Millenia System, Spectra Physics, Mountain View, CA). Images were acquired with Leica Confocal Software. Offline images were viewed with ImageJ (http://rsbweb.nih.gov/ij/). Images were aligned and corrected for movements created by breathing artifacts using a custom Matlab (Mathworks) program written in the laboratory. Images were deconvolved using Huygens deconvolution software (Scientific Volume Imaging, Hilversum, The Netherlands). Finally, axons were traced via the semi-automatic mode in Neuromantic (v1.6.3, http://www.rdg.ac.uk/neuromantic/), and voxel size was corrected with another custom Matlab (Mathworks) program. Boutons were then categorized depending on the fate of the axons on which they resided (stable, added, or retracted). Voxel size was corrected by a custom Matlab program.

## Abbreviations

AAV: adeno-associated virus
CMV: cytomegalovirus
eGFP: enhanced green fluorescent protein
eYFP: enhanced yellow fluorescent protein
